# The Vital Functions

**Published:** 1889-01

**Authors:** 


### THE VITAL FUNCTIONS.

It is well understood that the vital functions are more or less processes of combustion, and are subject to laws similar to those which regulate the burning of coal in our fire-places. We are apt to put on too much coal, or allow the fire to be smothered in ashes. The child pokes the fire from the top to make it burn faster ; but the wise man pokes it from below to rake out the ashes and allow free access of oxygen. And so it is with the functions of life, only that these being less understood, many a man acts in regard to them as a child does to the fire. The man thinks that his brain is not acting because he he has not supplied it with sufficient food. He take meat three times a day, and beef tea, to supply its wants, as he thinks, and puts in a poker to stir it up in the shape of a glass of sherry or a nip from the brandy bottle. And yet, all the time, his brain is suffering from accumulation of ash, and the more he continues to cram himself with food, and to supply himself with stimulants, the worse he ultimately becomes, just as the child's breaking the coal may cause a temporary blaze, but allows the fire to be smothered in ashes.

